# Long-Term Evaluation of a Ternary Mixture of Molten Salts in Solar Thermal Storage Systems: Impact on Thermophysical Properties and Corrosion

**DOI:** 10.3390/ma17164053

**Published:** 2024-08-15

**Authors:** Mauro Henríquez, Juan Carlos Reinoso-Burrows, Raúl Pastén, Carlos Soto, Carlos Duran, Douglas Olivares, Luis Guerreiro, José Miguel Cardemil, Felipe M. Galleguillos Madrid, Edward Fuentealba

**Affiliations:** 1 Iberian Centre for Research in Energy Storage (CIIAE), 10003 Cáceres, Spain; 2 Centro de Desarrollo Energético Antofagasta, Universidad de Antofagasta, Angamos 601, Antofagasta 1270300, Chile; juan.reinoso.burrows@ua.cl (J.C.R.-B.); carlos.soto@uantof.cl (C.S.); carlos.duran@uantof.cl (C.D.);; 3 ICT—Institute of Earth Sciences, University of Évora, 7000-308 Évora, Portugal; 4 Department of Mechanical and Metallurgical Engineering, Pontificia Universidad Católica de Chile, Vicuña Makenna 4860, Santiago 7820436, Chile; jmcardem@uc.cl

**Keywords:** molten salts, lithium nitrate, steel, corrosion

## Abstract

Solar thermal plants typically undergo trough operational cycles spanning between 20 and 25 years, highlighting the critical need for accurate assessments of long-term component evolution. Among these components, the heat storage media (molten salt) is crucial in plant design, as it significantly influences both the thermophysical properties of the working fluid and the corrosion of the steel components in thermal storage systems. Our research focused on evaluating the long-term effects of operating a low-melting-point ternary mixture consisting of 30 wt% LiNO_3_, 57 wt% KNO_3_, and 13 wt% NaNO_3_. The ternary mixture exhibited a melting point of 129 °C and thermal stability above 550 °C. Over 15,000 h, the heat capacity decreased from 1.794 to 1.409 J/g °C. Additionally, saline components such as CaCO_3_ and MgCO_3_, as well as lithium oxides (LiO and LiO_2_), were detected due to the separation of the ternary mixture. A 30,000 h exposure resulted in the formation of Fe_2_O_3_ and the presence of Cl, indicating prolonged interaction with the marine environment. This investigation highlights the necessity of analyzing properties under actual operating conditions to accurately predict the lifespan and select the appropriate materials for molten salt-based thermal storage systems.

## 1. Introduction

In the past decade, renewable energy, especially solar and wind power, has experienced significant growth worldwide. In that context, Chile has implemented a comprehensive decarbonization plan with the goal of shutting down 11 coal thermal power plants by 2024 and achieving complete closure of all 28 existing plants by 2040 [1,2]. Considering the reduction in coal generation, it is also reduced by the presence of synchronous generators that allow frequency control and grid stability. Therefore, such a scenario and the increasing installed capacity of variable renewable energy (VRE) sources, such as solar energy, constitute a significant technological challenge associated with their intermittent generation. To overcome this challenge, there is consideration for integrating thermal energy storage (TES) systems into concentrating solar power (CSP) plants, which allow exploiting the solar resource while also contributing to grid stability. Thus, the integration of CSP is foreseen to provide the required autonomy for a seamless transition to reliable VRE generation [3,4].

The storage system used in CSP plants consists of two tanks designed to store sensible heat, where molten salts are used as storage media. The material currently used in commercial CSP systems is a binary mixture called “Solar Salt”, which comprises a mixture of 60% NaNO_3_ by weight + 40% KNO_3_ by weight [5]. The operating principle employed in these plants revolves around sensible heat. This process entails elevating the temperature of a medium (usually solid or liquid) without inducing a phase change in the material. Consequently, the efficacy of this system is contingent upon factors such as specific heat capacity, density, thermal diffusivity, vapor pressure, and thermal conductivity [6].

Solar salt in CSP plants acts as a heat transfer fluid and thermal storage medium, offering advantages such as low cost, excellent heat transfer capabilities at high temperatures, high density, and stability. They are non-toxic, non-flammable, and have low vapor pressure and viscosity, which reduces pumping power requirements. Moreover, they are compatible with stainless steel alloys, commonly used for salt transport and storage in CSP systems [7,8].

The primary drawback of solar salt is its notably high melting point, which requires operating at elevated minimum temperatures, typically around 290 °C in commercial plants. Several investigations [9,10,11,12,13,14,15,16] have proposed alternative mixtures to address this limitation. For instance, studies have explored the addition of different proportions of lithium nitrate to solar salts to enhance their properties and expand their operational ranges in concentrated solar power (CSP) systems. Bradshaw and Siegel [14] conducted early research highlighting the benefits of incorporating lithium nitrate; however, they noted that its higher cost compared to potassium, calcium, and sodium nitrate could be a limiting factor. Additionally, they argued that this increased cost contributes to the limited use of lithium-based salts in commercial applications, along with the consideration of even more expensive additives such as AgNO_3_ and CsNO_3_.

In this context, several studies [15,16,17,18,19,20,21,22,23,24,25] have investigated the composition and thermal properties of ternary salt mixtures composed of LiNO_3_, NaNO_3_, and KNO_3_, using experimental and theoretical methods to determine their optimal characteristics for thermal storage. These studies share the methodology of employing techniques such as differential scanning calorimetry (DSC), thermogravimetric analysis (TGA), and viscosity to validate theoretical findings, highlighting the consistency and precision of the obtained results. They all emphasize the importance of the composition and purity of the components in determining the thermal properties and stability of ternary salt mixtures, evidencing their viability as thermal storage media in solar applications. This specific composition demonstrated viscosities closely comparable to those of solar salt, registering at 3.3 cP at 150 °C and 2.8 cP at 300 °C.

One of the complexities of thermal energy storage (TES) systems using molten salts is the design of the tanks that must contain this material at high temperatures, depending on the solar thermal technology employed. In the parabolic trough technology, two operational tanks are used: the cold tank, which operates at 290 °C, and the hot tank, which reaches approximately 390 °C. The temperatures are higher for tower technologies, with the cold tank operating at 290 °C and the hot tank at 565 °C. Generally, the cold tank is constructed with the American Society for Testing and Materials standard ASTM A-516 Gr.70 carbon steel [26,27], while the hot tank is made from austenitic stainless steels (Fe–Cr–Ni alloys) such as AISI 316L, AISI 304, AISI 347H, or AISI 321H [6,8], which have a high chromium content to increase corrosion resistance.

Corrosion in these systems, driven by thermodynamic processes, increases with the temperature of the molten salts. Therefore, the choice of tank material must consider the storage temperature. The most common forms of corrosion in these contexts are high-temperature corrosion, localized corrosion, mechanically assisted corrosion, and flow-accelerated corrosion [28].

Regarding the corrosion process of steels in contact with nitrate salts, Mallco et al. [29] determined that iron reacts with O_2_ and generates products such as FeO, FeO_2_, Fe_3_O_3_, and Fe_2_O_3_, which generate a layer of metallic oxide. In addition, it was identified that the species generated by the reaction between the O^2−^ due to the dissociation of NO_3_ ions in the electrolyte is given by the following equations:

FeO + O^2−^ = FeO_2_^2−^ (1)

Cr_2_O_3_ + 3O^2−^ = Cr_2_O_4_^2−^ (2)

NiO + O^2−^ = NiO_2_^2−^ (3)

Cheng et al. [30] conducted additional high-temperature corrosion tests on various carbon steels in contact with a Li/Na/K nitrate mixture. The results showed that LiNO_3_ promotes corrosion, and chromium (Cr) enhances corrosion resistance by forming an internal protective layer of (Fe,Cr)_3_O_4_ directly on the surface of the steel (4). This layer acts as a barrier that inhibits iron diffusion from the steel substrate, thereby slowing down the corrosion process, along with an external layer of LiFeO_2_ (5), a finding supported by another study [27,31].

3Fe^2+^ + 4Cr_2_O_3_ → 3(Fe,Cr)_3_O_4_ + 2Cr^3+^ (4)

Fe^3+^ + Li^+^ + 2O^2−^ → LiFeO_2_ (5)

6Fe_2_O_3_ + 2Li^+^ → 2LiFe_5_O_8_ + 2Fe^3+^ + 2O^2−^ (6)

LiFe_5_O_8_ + 3Li^+^ → 4LiFeO_2_ + Fe^3+^ (7)

**Figure 1:**
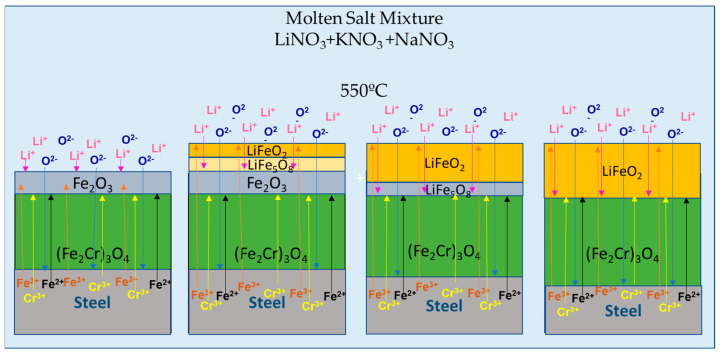
Formation of corrosion products of steel and a ternary mixture of Li/Na/K nitrates. Adapted from [30].

It was determined that this external layer forms at the interface between Fe_2_O_3_ and the mixed nitrates Li/Na/K. As Li diffusion continues, new interfaces are formed, such as LiFe_5_O_8_ and Fe_2_O_3_, which are consumed in the initial stages of corrosion to form LiFeO_2_ and Fe^3+^ according to Reactions (6) and (7). These chemical processes are represented more clearly in Figure 1.

Previously, various studies have been conducted on the behavior of the thermal and corrosive properties of steel materials at the laboratory level, but not in larger-scale tests. We argue that relying solely on preoperational data can introduce inaccuracies that affect design decisions, especially in molten salts, which undergo thermal property changes over time. This consideration becomes particularly crucial for high-temperature thermal storage applications. In the present study, we aim to address this gap by evaluating the long-term thermophysical properties of the Li/Na/K nitrate ternary mixture and the corrosive behavior of the low-alloy salt bomb steel under conditions similar to the actual operating conditions within a molten salt tank.

## 2. Materials and Methods

### 2.1. Materials

**Figure 2:**
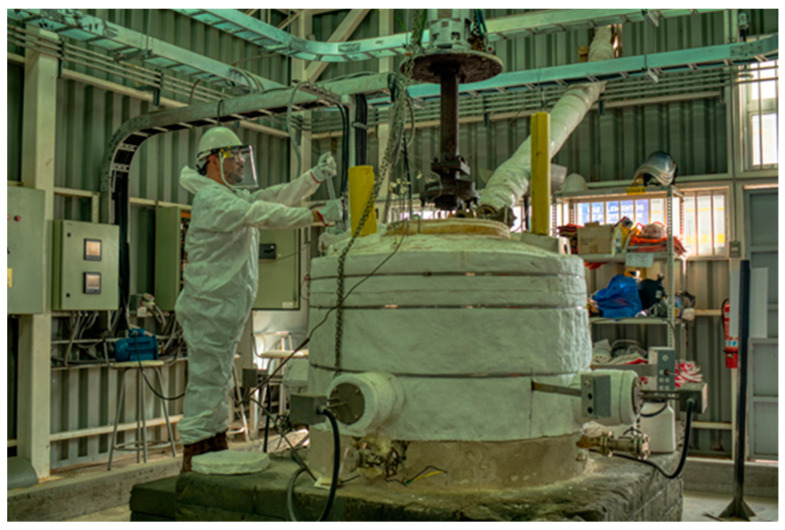
Molten salt pilot plant at the University of Antofagasta, Chile.

The ternary mixture of Li/Na/K nitrates was prepared using sodium nitrate (NaNO_3_, 99.5%) and potassium nitrate (KNO_3_, 99.5%), both supplied by SQM (Antofagasta, Chile), and nitrate lithium (LiNO_3_, 99%) from Todini Chemical (Navi Mumbai, India). A 316L grade stainless steel tank loaded with 700 kg of the mixture was used for the experimental tests. The tank, shown in Figure 2, was equipped with four electrical resistances to provide the heat necessary to heat the salts, temperature sensors in different positions inside and outside the tank, a high-temperature vertical pump, electrical tracing, and a temperature control and monitoring system.

The thermophysical properties of the ternary salt were assessed at the initial stage (t = 0 h) and subsequently after 15,000 h of operation in triplicate in the Antofagasta laboratory in Antofagasta, Chile. The melting point and heat capacity were determined using a DSC and modulated DSC study, respectively, on a TA Instruments Q20 calorimeter. Each sample, weighing between 10 and 12 mg, was placed in hermetically sealed aluminum crucibles. Tests were conducted from 30 °C to 400 °C. This involved an initial heating ramp at a rate of 10 °C/min, followed by a cooling cycle at the same speed [32,33].

The mixtures’ viscosity was evaluated with a Brookfield DV-III viscometer, with readings taken at temperatures of 150, 175, 200, and 300 °C. For these measurements, a Brookfield 21S SC4-31 stainless steel (Antofagasta Laboratory, Antofagasta, Chile) spindle was used, adapted to the viscosity range of the molten salt mixture. Thermogravimetric analysis (TGA) was performed using a Mettler Toledo TGA-DSC 1 LF/894 STARe (Antofagasta Laboratory, Antofagasta, Chile) between a temperature of 25 °C and 600 °C at 10 K min^−1^. The samples were weighed and maintained a mass of between 10 and 18 mg, and they were placed in the aluminum crucibles for TGA analysis.

### 2.2. Corrosion Process

This process underwent evaluation over a period exceeding 30,000 h. involving contact between the molten salts and the molten salt pump. A mineralogical characterization of crystalline species was conducted using X-ray diffractometry (XRD) (Antofagasta Laboratory, Antofagasta, Chile) to observe the evolution over time of the crystalline phases present in the ternary salts. The analysis employed a PANalytical X’Pert Pro MPD X-ray diffractometer (Antofagasta Laboratory, Antofagasta, Chile) with Cu-Kα radiation, scanning a 2θ angle range between 15° and 80° at a speed of 0.014° s^−1^. To enhance the reliability of the analysis, measurements were performed in triplicate.

The microstructure analyses of the samples were performed by field emission scanning electron microscopy (FE-SEM) (Antofagasta Laboratory, Antofagasta, Chile) and energy dispersive spectroscopy (EDS) (Antofagasta Laboratory, Antofagasta, Chile). Analyses were performed using a Zeiss EVO MA 10 microscope (Zeiss, Oberkochen, Germany) and a Hitachi SU-5000 model (Tokyo, Japan).

## 3. Results and Discussion

### 3.1. Thermophysical Properties

**Figure 3:**
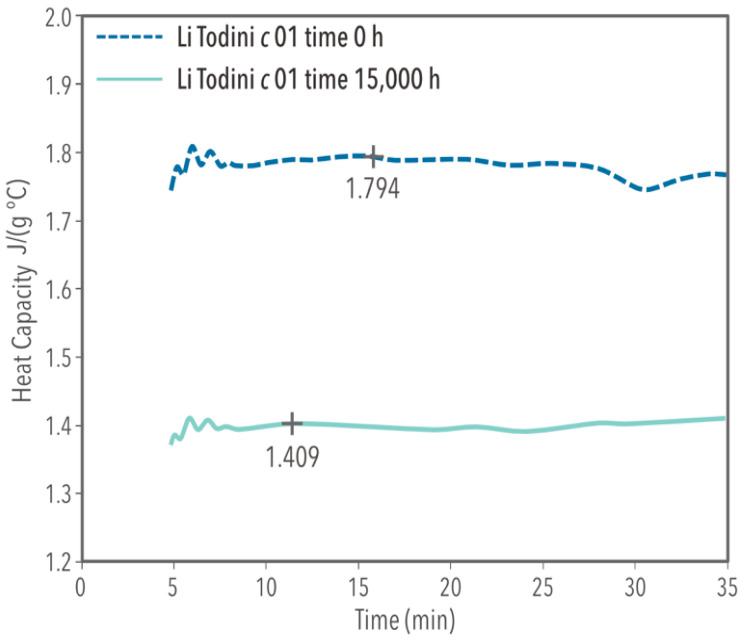
MDSC test of ternary mixture at 390 °C, at time t = 0 h and t = 15,000 h.

**Figure 4:**
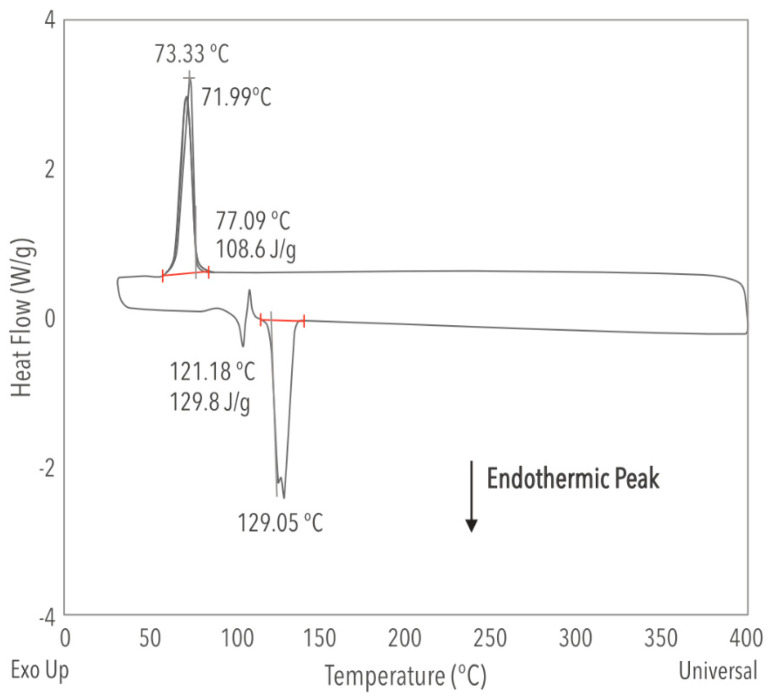
DSC analysis of ternary mixture of Li/Na/K at t = 15,000 h.

At the testing’s early stage (t = 0 h), the ternary molten salt mixture demonstrated a melting point of 124 °C and a heat capacity of 1.794 J/(g °C) at 390 °C. For t = 15,000 h, the heat capacity (Figure 3) was 1.409 J/(g °C) at 390 °C, the melting point (endothermic) at 129 °C, and the solidification point when cooling down (exothermic) at 73.33 °C are shown in Figure 4. During heating, a transition peak appears prior to melting, indicating a solid-solid transformation in the mixture [19]; however, during the cooling process, we observed no transition peak. Incorporating lithium nitrate reduces the minimum operation temperature by almost 90 °C compared to solar salt. In contrast, the heat capacity behavior depends on the operating stage: At t = 0 h, it was 20% higher than the conventional solar salt, but after 15,000 h of operation, it decreased by approximately 6%. Table 1 summarizes the thermal properties of the mixture at time t = 0 h and t = 15,000 h.

**Figure 5:**
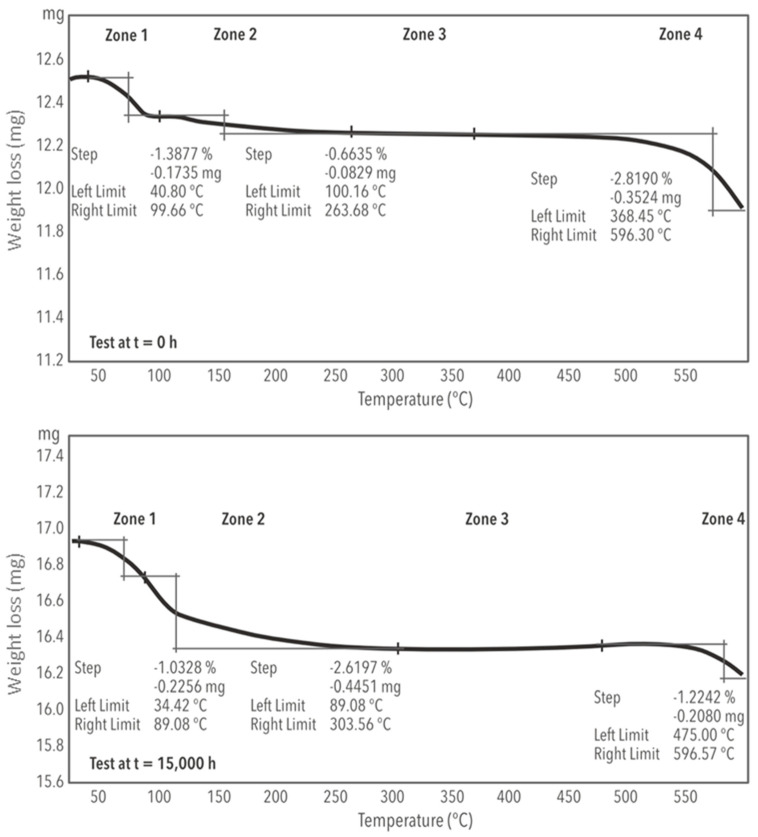
Thermal stability of ternary mixture of Li/Na/K with 99% LiNO_3_ purity at time t = 0 h (**up**) and t = 15,000 h (**bottom**).

The thermal stability results for both exposure times of the samples are shown in Figure 5, where different zones are identified according to Henriquez et al. [20]. In the initial zones, mass loss due to supramolecular and intramolecular water is observed, followed by a much more stable zone without mass loss, and finally, a last zone where the ternary mixture is decomposed.

Table 2 compares the weight loss for the two exposure times studied. The weight loss in zone 1 is very similar for the two samples. In zone 2, the loss of mass for the time of 15,000 h quadruples from the time of 0 h, from 0.66% to 2.62%, because during this exposure time, some impurities are released from the salt that have been formed during the 15,000 h. In zone 3, a more stable area is observed, with a broader temperature differential, reaching 475 °C without mass loss. Zone 4, with a 596 °C maximum temperature, presents an almost negligible mass loss percentage at the end of the cycle compared to the initial value.

The viscosities obtained for the lithium mixture at t = 0 h and t = 15,000 h, values measured below 10 cP (Table 3) for both exposure times at 230 °C, are shown in Table 3. The binary solar salt showed lower viscosities, regardless of the temperature range. The ternary mixture’s viscosity varied inversely proportional to the temperature, ranging from 20 cP to 8 cP between 150 °C and 300 °C, respectively. These results agree with data from the literature [24,25,34].

### 3.2. Corrosion Process and Decomposition of the Ternary Mixture

**Figure 6:**
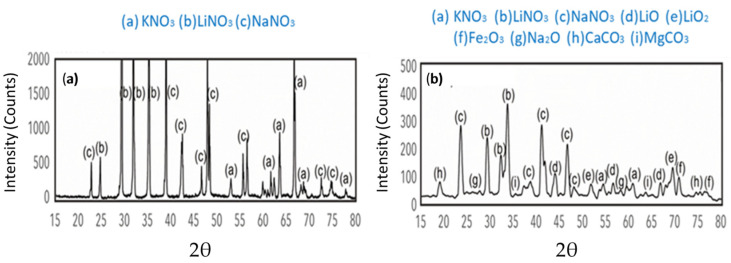
X-ray diffraction analysis of the ternary mixture at different exposure times between the salts and the steels (**a**) at 0 h, (**b**) after 30,000 h.

The ternary mixture was exposed to different steel equipment at 400 °C for 30,000 h. An X-ray diffraction analysis was carried out to evaluate the changes in composition caused by the corrosion of the steel. The results of this analysis are shown in Figure 6. In Figure 6a, at t = 0, which corresponds to the ternary mixture, no internal oxidation is observed upon contact with the pump’s suction surface, and KNO_3_, LiNO_3_, and NaNO_3_ are present. In contrast, Figure 6b shows morphological damage on the pump’s surface at high temperatures. This is evident by the formation of different products in the ternary mixture during long exposure periods, detecting the presence of LiO, LiO_2_, Fe_2_O_3_, Na_2_O, CaCO_3_, and MgCO_3_. It was possible to identify small peaks of Fe_2_O_3_ that correspond to the corrosion interfaces formed during the diffusion process of iron from the steel and oxygen from the outside [30]. The presence of lithium oxides was consistent with findings by Wang et al. [35], who identified LiO and LiO_2_. This indicates that LiNO_3_ is the unstable species and the component responsible for the weight loss in the ternary mixture. The formation of CaCO_3_ and MgCO_3_ in the analyses may be due to the interaction of impurities with atmospheric gases, where oxygen ions react with CO_2_ to generate [7,14]. The problem with the formation of these types of elements is that they are insoluble and can cause operational problems by clogging or eroding the pipes of the thermal storage circuit.

The presence of Na_2_O corresponds to the decomposition of NaNO_3_ [35]. However, the signal for this compound is small, while the signal for NaNO_3_ is higher and clearer, which confirms the thermal stability of this compound despite prolonged exposure to the salts [36,37].

**Figure 7:**
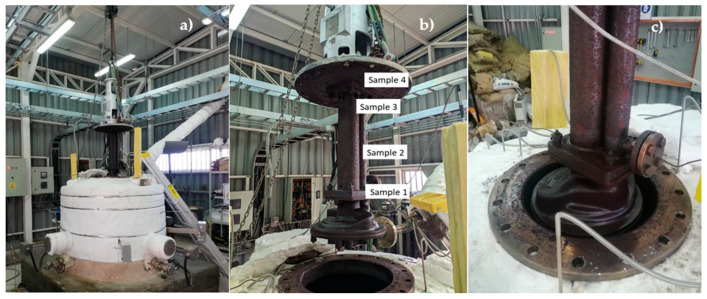
Sample distribution on the pump shaft.

In Figure 7, it is possible to see the corrosion suffered by the pump. Sample 1 corresponds to the region where the pump impeller sample was obtained. The composition of all the samples can be seen in Table 4. It consists of ASTM A 217 grade WC6 steel, a low-alloy steel containing small amounts of other elements besides iron and carbon, which were in direct contact with the ternary molten salts. On the other hand, samples 2, 3, and 4 represent areas that were not in direct contact with the ternary mixture but were inside the tank, exposed to high temperatures, and influenced by hot air and nitrous oxide gases released from the ternary mixture. Figure 7a provides a view of the pump corrosion inside the molten salt pilot tank. Figure 7b provides a view of the four points where the different corrosion samples were collected in the pump, finally, Figure 7c provides a detailed view of sample 1, which was in contact with the ternary mixture inside the salt tank. melted.

**Figure 8:**
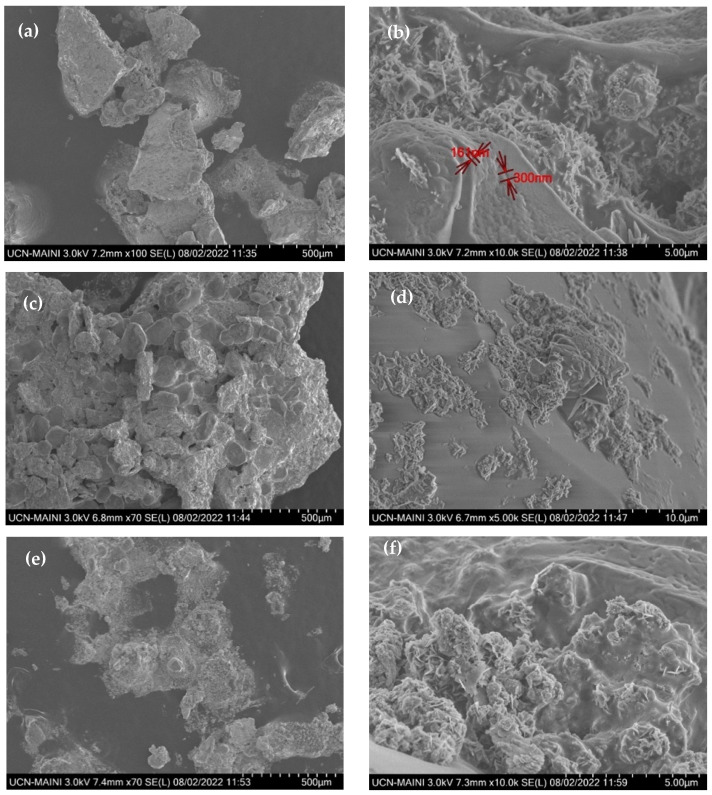
Corrosion product from the contact of the steel of the pump shaft with the hot air and the release of nitrous oxide gases. Sample 2 (**a**,**b**), sample 3 (**c**,**d**) and sample 4 (**e**,**f**).

Figure 8a,b corresponds to sample 2, Figure 8c,d corresponds to sample 3, and finally, Figure 8e,f corresponds to sample 4. These samples were not in direct contact with the molten salt. In these cases, the formation of adherent oxide on the steel surface can be observed. This is attributed to the contact of the steel of the pump shaft with the hot air and the release of nitrous oxide gases generated by the ternary mixing. In all the cases mentioned, evident material damage is observed.

**Figure 9:**
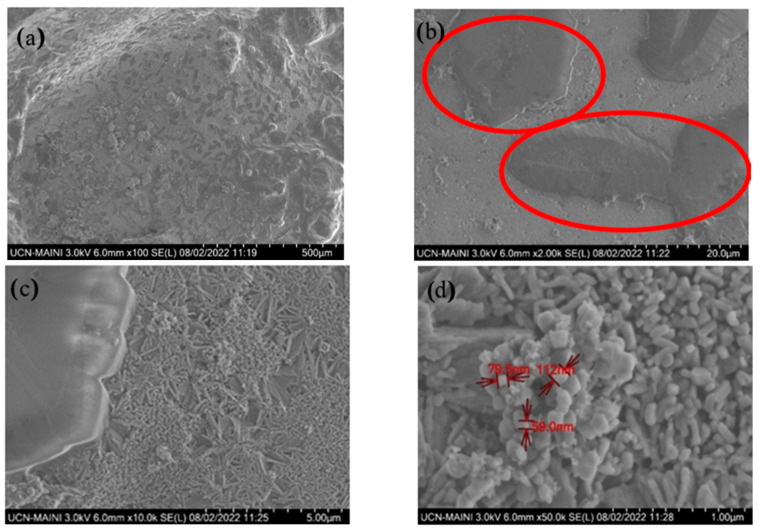
Corrosion product from the shaft of the molten salt pump. (**a**–**d**) Sample 1.

Figure 9 shows the FE-SEM analyses carried out on sample 1, which was in direct contact with the molten salt. It is observed that the material has deteriorated, with a dense crystalline morphology that corresponds to corrosion flakes and the presence of evident cracks in Figure 9b (highlighted in red), which shows the chemical process that occurs on the surface of the steel. This observation is further supported by Figure 9c,d, where the presence of prism-shaped salt with sizes varying from 55 to 112 nm is observed.

Molten ternary salts operating in a liquid state at high temperatures (400 °C) have been found to be highly corrosive in various studies [37,40], which is also confirmed by our analyses. These extreme and harsh conditions accelerate the degradation of pump components, affecting the overall project’s profitability.

**Figure 10:**
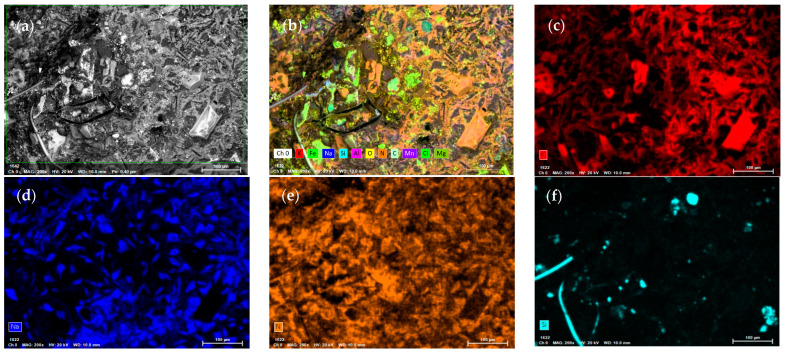

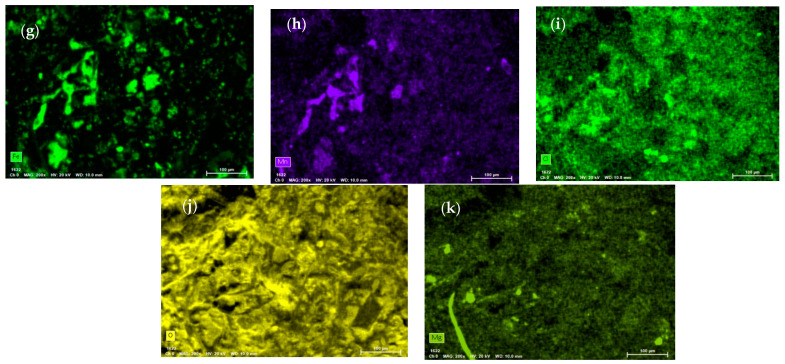
EDS mapping of corrosion products of the pump shaft that was in contact with molten ternary salts during a 30,000 h operation. (**a**) random section of the selected sample, (**b**) presence of different elements in the selected area, (**c**) K, (**d**) Na, (**e**) Ni, (**f**) Si, (**g**) Fe, (**h**) Mn, (**i**) Cl, (**j**) O and (**k**) Mg.

The EDS results show the following: Figure 10a corresponds to a random section of the sample selected for analysis. The results obtained in this random section show the presence of iron oxides (Figure 10b,g), confirming the presence of hematite detected through X-ray diffraction analysis. The presence of Si and Mn (0.34% by weight) and Mn (0.18% by weight) were also identified, which are part of the typical composition of ASTM A217 Grade WC6 steel (Figure 10f,h). The Mg components (0.07 wt%) are considered impurities of the ternary mixture. Finally, the presence of chlorine in low amounts, at 0.01 wt% (Figure 9i), is detected. This element is attributed to the marine atmosphere, as the laboratory is located in Antofagasta, approximately 200 m from the sea [41,42]. At high temperatures, this ion significantly accelerates corrosion due to its extensive dispersion on the suction surface of the pump. Other elements detected include potassium 12.81 *w*/*w*% (Figure 10c), sodium 7.66 *w*/*w*% (Figure 10d), and nitrogen 19.98 *w*/*w*% (Figure 10e).

Although the steel shows evident corrosion with the formation of multiple oxide layers on its surface, there is no visible degradation or loss of surface material. However, the degradation that occurred could only be detected through X-ray diffraction, despite prolonged exposure to high temperatures in the ternary salt. On the other hand, although it was not possible to perform a mass loss analysis before and after this entire process, it should be noted that the increase in metal losses is directly proportional to the square root of time, which indicates that the growth of the surface deposits follows a self-limiting process. This process is controlled by the diffusion of one of the chemical species that make up the deposits. Furthermore, it should be noted that if the temperature continues to increase above 400 °C, the corrosion rate can accelerate [43,44]. and could directly affect the material, aggravating stress corrosion cracking (SCC) [45,46].

Through FE-SEM and X-ray diffraction, the morphological damage to the microstructure of this type of low-alloy steel in the pump suction can be seen due to contact for a long period with the ternary mixture. Knowing the changes that occur over time, such as the formation of different corrosion products and the effects it causes on materials, shows us how necessary it is to carry out this type of analysis for conditions very similar to real ones, to project the operational life and determine what type of material would be necessary to use together with the ternary mixture of Li/Na/K nitrates as thermal storage systems.

## 4. Conclusions

In this research, we investigated the effect of long-term operation on the thermophysical properties of a molten salt ternary mixture and the corrosion process that occurs within the metallic surface of the pump. The main conclusions of this work are the following:

(1) After 15,000 h, the heat capacity decreased from 1.794 to 1.409 J g^−1^ °C^−1^. We found this to be the property most sensitive to long-term usage, which might be especially relevant if our mixture is utilized in thermal energy storage.
(2) The results for the immersed salt inside the salt tank at time t = 0 and t = 15,000 h showed that the ternary mixture offers a low melting point of 124 and 129 °C, respectively, which is lower than the conventional solar salt. The latter could potentially reduce the CSP plant’s operating gap and would allow for maintaining a lower operating temperature with evident cost savings.
(3) For both times, its decomposition temperature is higher than 590 °C, considering that the weight loss remained within 3%.
(4) The viscosity decreases as the temperature increases, and above 200 °C, the viscosity values are below 12 cP, which approximates the viscosity range of the solar salt (4 to 7 cP).
(5) After 30,000 h, we detected saline components, such as CaCO_3_ and MgCO_3_, produced by the decomposition of the ternary mixture. These can generate clogging problems in the pipes due to the formation of insoluble solids. We also identified lithium oxides (LiO and LiO_2_) produced by the decomposition of the unstable species of the ternary mixture as LiNO_3_.
(6) After 30,000 h of contact between the ternary mixture of nitrates and various components, such as the internal surface of the steel tank and the suction of the molten salt pump, Fe_2_O_3_ was identified as a corrosion product. Additionally, Cl was detected, probably from the local marine atmosphere, where salts can be transported in the air from nearby seas. These findings highlighted the relevance of the interaction of these components with the liquid salt mixture.
(7) Due to the low melting point, viscosity, and heat capacity, the ternary mixture could be an excellent substitute for solar salt for thermal storage applications. However, lithium currently has its market in electrochemical batteries, which is undergoing vigorous development, and as prices are very volatile, it could not compete with solar salt.

## Figures and Tables

**Table 1:** Thermal results obtained in ternary mixture and solar salt.

Mixture	Melting Point (°C)	Decomposition Temperature (°C)	Solidification Point (°C)	Heat Capacity (J g^−1^ °C^−1^)	(% wt) Heat Capacity/Heat Capacity Solar Salt
30 wt% LiNO_3_ + 13 wt% NaNO_3_ + 57 wt% KNO_3_ Todini (99% Li time 0 h)	125	596.3	76.05	1.794	+21.6
30 wt% LiNO_3_ + 13 wt% NaNO_3_ + 57 wt% KNO_3_ Todini (99% Li time 15,000 h)	129	596.57	73.33	1.409	−6.0
Solar salt	223	565	-	1.50	0

**Table 2:** Thermal stability zones for the ternary mixture 30%wt. LiNO_3_ + 13%wt. NaNO_3_ + 57%wt. KNO_3_ at time t = 0 h and time t = 15,000 h.

	Zone 1 (Supramolecular Water Losses)		Zone 2 (Intramolecular Water Losses)		Zone 3 (Stability)		Zone 4 (Decomposition)		Total Weight Loss (wt%)
Time	T °C	Weight Loss (wt%)	T °C	Weight Loss (wt%)	T °C	∆T	T °C	Weight Loss (1–3 wt%)	
0 h	40–100	1.39%	100–264	0.664%	264–368	104	368–596	2.84%	4.3
15,000 h	34–89	1.32%	89–303	2.62%	303–475	172	475–596	1.22%	5.2

**Table 3:** Viscosity values for ternary mixtures.

T (°C)	Viscosity (cP) LiNO_3_ 99% (t = 0 h)	Viscosity (cP) LiNO_3_ 99% (t = 15,000 h)	Viscosity (cP) Solar Salt
150	20.4	20.53	-
170	13.81	14.25	-
200	9.78	12.20	6.7
230	8.74	9.22	5.51
260	8.55	8.76	4.83
300	7.13	7.2	4.68

**Table 4:** Composition of steel material (ASTM A 217 Grade WC 6) [38,39].

ASTM A 217	%C	%Si	%Mn	%P	%S	%Cr	%Mo	%Ni	%Cu
	0.05–0.2	0.6	0.5–0.8	0.035	0.035	1–1.5	0.45–0.65	0.5	0.5

## Data Availability

The original contributions presented in the study are included in the article, further inquiries can be directed to the corresponding author.
